# Voltammetric Study of Some 3-Aryl-quinoxaline-2-carbonitrile 1,4-di-*N*-oxide Derivatives with Anti-Tumor Activities

**DOI:** 10.3390/molecules22091442

**Published:** 2017-08-31

**Authors:** Eric M. Miller, Qing Xia, Mariah E. Cella, Austin W. Nenninger, Monica N. Mruzik, Krystina A. Brillos-Monia, Yong Zhou Hu, Rong Sheng, Christina M. Ragain, Philip W. Crawford

**Affiliations:** 1 Department of Chemistry, Southeast Missouri State University, Cape Girardeau, MO 63701, USA; emmiller1s@semo.edu (E.M.M.); tomariahcella@gmail.com (M.E.C.); anenninger@health.usf.edu (A.W.N.); mnmruzik@semo.edu (M.N.M.); kbrillos8@gmail.com (K.A.B.-M.); cragain@semo.edu (C.M.R.); 2 Department of Pharmacology, Ningbo College of Health Sciences, No. 51, Xuefu Road, Yinzhou, Ningbo 315000, China; sunnyxq@zju.edu.cn; 3 College of Pharmaceutical Sciences, Zhejiang University, Hangzhou 310058, China; huyz@zju.edu.cn (Y.Z.H.); shengr@zju.edu.cn (R.S.)

**Keywords:** quinoxaline-di-*N*-oxide derivatives, voltammetry, anti-tumor, reduction potential, experimental and computational

## Abstract

The electrochemical properties of twenty 3-aryl-quinoxaline-2-carbonitrile 1,4-di-*N*-oxide derivatives with varying degrees of cytotoxic activity were investigated in dimethylformamide (DMF) using cyclic voltammetry and first derivative cyclic voltammetry. With one exception, the first reduction of these compounds was found to be reversible or quasireversible and is attributed to reduction of the *N*-oxide moiety to form a radical anion. The second reduction of the diazine ring was found to be irreversible. Compounds containing a nitro group on the 3-phenyl ring also exhibited a reduction process that may be attributed to that group. There was good correlation between molecular structure and reduction potential, with reduction being facilitated by an enhanced net positive charge at the electroactive site created by electron withdrawing substituents. Additionally, the reduction potential was calculated using two common basis sets, 6-31g and lanl2dz, for five of the test molecules. There was a strong correlation between the computational data and the experimental data, with the exception of the derivative containing the nitro functionality. No relationship between the experimentally measured reduction potentials and reported cytotoxic activities was evident upon comparison of the data.

## 1. Introduction

Quinoxaline 1,4-di-*N*-oxide derivatives have been the subject of worldwide interest in medicinal chemistry for a number of years due to their broad range of biological properties [1,2,3], as a variety of quinoxaline 1,4-dioxide derivatives have been reported to possess varying degrees of antibacterial [4,5], antimycobacterial [6], antitrypanocidal [7], antimalarial [8], anti-Chagas [9,10], antifungal [5,11], antioxidant/anti-inflammatory [12], and anticancer [13,14,15] activities. As a result, a large number of new quinoxaline 1,4-dioxides are being synthesized and their biological properties tested each year. One important feature for their biological activities is the presence of both *N*-oxide functional groups in the pyrazine ring of the basic quinoxaline structure. *N*-oxidation generally enhances the scope and level of their biological properties [1,2,16], and may be important for bioreduction [10]. It is well-known that some quinoxaline 1,4-di-*N*-oxide derivatives undergo bioreduction under hypoxic conditions, leading to the formation of a radical capable of cleaving DNA [2,3]. The latter may occur via direct abstraction of hydrogen atoms from DNA or production of DNA-cleaving hydroxyl radicals, both mechanisms introducing oxidative stress within the target cells.

Likewise, *N*-oxidation enhances the ease of reduction of the quinoxaline ring [17]. Previous studies have shown that there is a relationship in some cases between the ease of reduction for certain homologous series of quinoxaline 1,4-di-*N*-oxides and their reported biological activities [10,17,18,19,20].

Because their redox properties can influence their biological activities, electrochemical studies of quinoxaline 1,4-di-*N*-oxide systems may help in understanding their mechanisms of action, as well as in the design of new drugs. Recently, a series of 3-aryl-quinoxaline-2-carbonitrile 1,4-di-*N*-oxide derivatives (Table 1) were synthesized and evaluated for their cytotoxic activities [21]. Many of these compounds displayed more potent hypoxic cytotoxic activity than 3-aminobenzotriazine-1,4-dioxide (TPZ) and 3-amino-2-quinoxalinecarbonitrile 1,4-di-*N*-oxide (TX-402), both of which display promising anticancer activities. TPZ has been shown to undergo bioreductive activation leading to the formation of radical species that cause DNA damage [21]. The goal of this present work was to study the general electrochemical characteristics of these 3-aryl-quinoxaline-2-carbonitrile 1,4-di-*N*-oxide derivatives under nonaqueous conditions and to investigate potential relationships between their redox properties, structures, and reported biological activities. In addition, we investigated the ability to calculate the reduction potential for five test molecules (compounds **1a**–**1e**) using standard computational methods and basis sets. Computational chemistry may provide a way to screen potential future molecules in order to direct synthesis of new and novel derivatives [22].

## 2. Results and Discussion

### 2.1. Electrochemical Behavior

**Figure 1:**
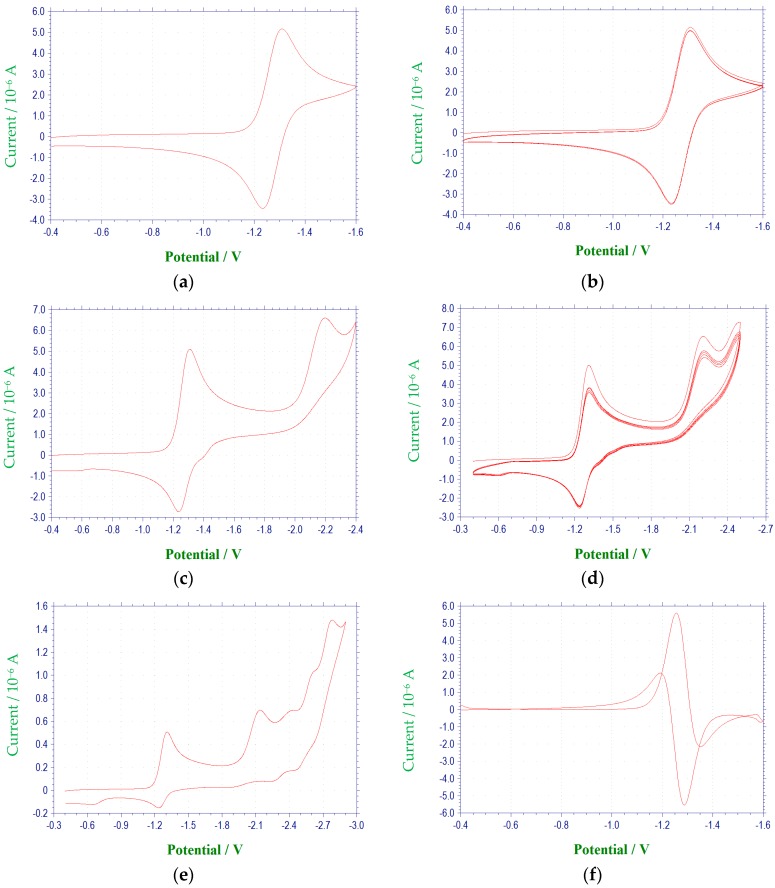

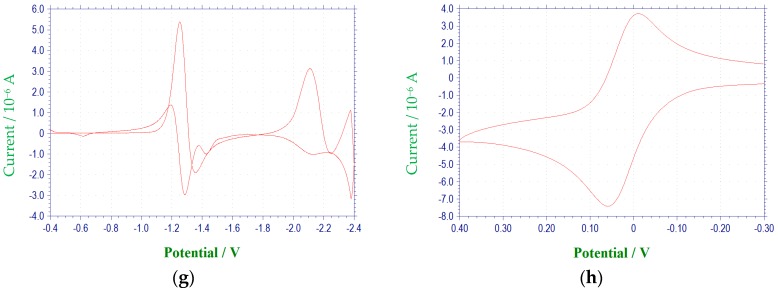
Cyclic voltammetric reduction of compound **1a** in DMF at 100 mV/s (E vs. (Ag/AgNO_3_)/V): (**a**) single scan between −0.4 and −1.6 V; (**b**) multiple scans between −0.4 and −1.6 V; (**c**) single scan between −0.4 to −2.4 V; (**d**) multiple scans between −0.4 and −2.4 V; (**e**) single scan between −0.4 and −2.9 V; (**f**) first derivative cyclic voltammogram between −0.4 and −1.6 V; (**g**) first derivative cyclic voltammogram between −0.4 and −2.4 V; (**h**) Cyclic voltammogram for the ferrocene redox couple used as a reference for reporting peak potentials.

**Figure 2:**
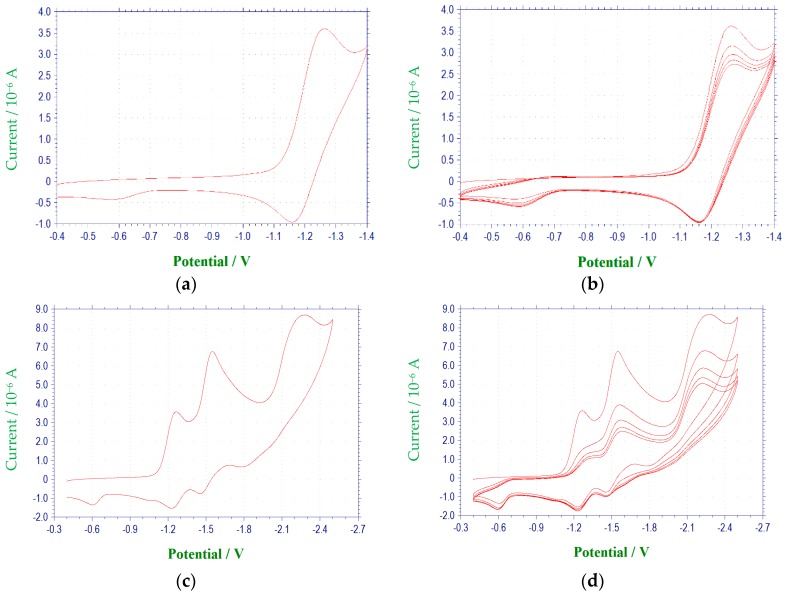

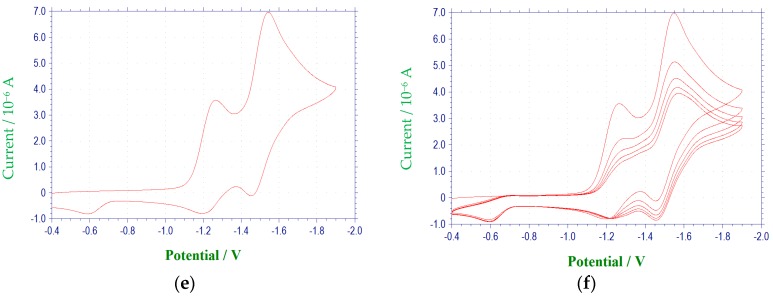
Cyclic voltammetric reduction of compound **1e** in DMF at 100 mV/s (E vs. (Ag/AgNO_3_)/V): (**a**) single scan between −0.4 and −1.4 V; (**b**) multiple scans between −0.4 and −1.4 V; (**c**) single scan between −0.4 and −2.5 V; (**d**) multiple scans between −0.4 and −2.5 V; (**e**) single scan between −0.4 to −1.9 V, (**f**) multiple scans between −0.4 and −1.9 V.

The compounds included in this study are 3-aryl-quinoxaline-2-carbonitrile 1,4-di-*N*-oxide derivatives (Table 1) that have been evaluated for their biological activities as hypoxic selective anti-tumor agents [21]. The derivatives possess varying substituents in the 3 and 7 positions of the basic quinoxaline structure. The redox properties of these substances were investigated via cyclic voltammetry and first derivative cyclic voltammetry in DMF using a platinum disc-working electrode. The reductions observed for these compounds were found to be diffusion controlled based on current functions that were relatively independent of scan rate and linearity in the plots of cathodic peak current versus the square root of scan rate [23,24]. Electrochemical data for scans obtained at 100 mV/s are summarized in Table 2 and Table 3, and representative voltammograms are shown in Figure 1 and Figure 2. All redox potentials reported in this study are relative to the (Fc/Fc^+^) redox couple.

**Figure 3:**
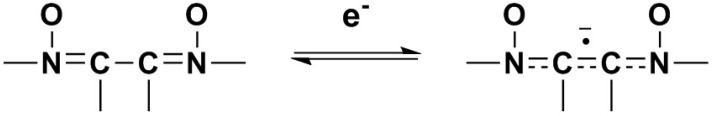
One electron reduction of the di-*N*-oxide structure to a radical anion.

The electrochemical characteristics of a number of quinoxaline di-*N*-oxide derivatives have been reported previously [10,17,18,19,20,25,26,27]. The first voltammetric wave observed, representing the reduction of a *N*-oxide functionality to form a radical anion [27], was reversible or quasireversible for all derivatives studied, with the exception of **3c**. (Figure 3) E_1/2_ values for this reduction process ranged from −1.154 V to −1.333 V. Values of ΔE_p_ and E_pc_ − E_1/2_ for this wave were typically greater than the theoretical values of 57 mV and −28.5 mV [23,24], respectively, for a reversible, one electron reduction. Estimates of the number of electrons involved in this reduction process based on the observed values for ΔE_p_ and E_pc_ − E_1/2_ verify the one electron nature of this reduction. Likewise, calculated i_pa_/i_pc_ ratios for derivatives **1a**, **1c**, **1d**, **2a**–**2d**, **3a**, **3b**, **3d** and **4a**–**4d** were close to one at all scan rates, indicating that relatively stable reduction products were formed within the time frame of the experiment [23]. For compounds **1b**, **1e**, **2e**, **3e** and **4e**, current ratios were significantly less than one, i.e., 0.2 to 0.3, indicating kinetic or other complications [23]. For compound **3c**, the first reduction process was irreversible with E_pc_ = −1.401 V. Although the first reduction for quinoxaline di-*N*-oxide derivatives is typically reversible or quasireversible in aprotic solvents, examples of irreversibility in this process for some quinoxaline di-*N*-oxides has been noted previously under conditions similar to those used in this study, i.e., voltammetry at a platinum working electrode in DMF [17].

The relationship between quinoxaline structure and reduction potential may be observed via examination of the data in Table 2 and Table 3. Replacement of a H atom in the 3-/4-position of the 3-aryl group or 7-position of the quinoxaline ring with an electron donating group which increases the electron density in the conjugated system generally resulted in a negative shift in potential (E_1/2_ or E_pc_), making the reduction more difficult (cf. **1a** vs. **1b**, **1a** vs. **2a**, **1a** vs. **3a**). Replacement of a H atom in those same positions with an electron withdrawing group which removes electron density from the conjugated system resulted in a more facile reduction by shifting the potential in the positive direction (cf. **1a** vs. **1c**, **1a** vs. **1d**, **1a** vs. **1e**, and **1a** vs. **4a**). Thus, the change in electron density that occurs with a change in substituent is transmitted through the conjugated system to the electroactive heterocyclic ring.

**Figure 4:**
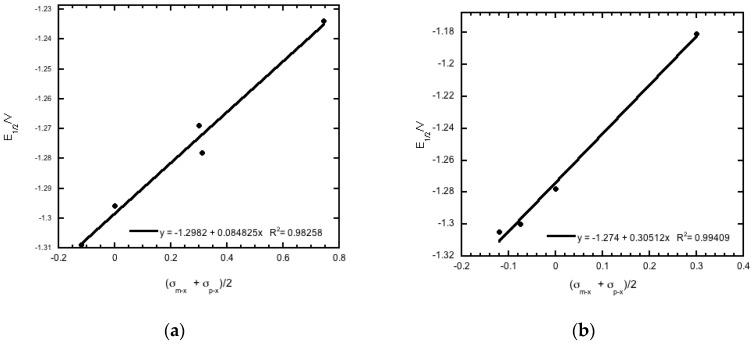
Plot of (**a**) (σ_m−*x*_ + σ_p−*x*_)/2 for substituent group R_2_ versus half-wave potential (E_1/2_ vs. Fc/Fc^+^) for compounds **1a**–**1e**, and (**b**) (σ_m−*x*_ + σ_p−*x*_)/2 for substituent group R_1_ versus half-wave potential (E_1/2_ vs. Fc/Fc^+^) for compounds **1d**, **2d**, **3d**, and **4d**. Hammett substituent constant values are taken from reference [29].

The 20 compounds studied can be broken down into different analogues based on structure, as evident from Table 1. The reduction potentials for the various derivatives within each analogous series fit the modified Hammett equation, ΔE_1/2_ = ρ_π,R_σ_*x*_ [28] with correlation coefficients that ranged from 0.92 to 0.99. In this equation, ρ_π,R_ is a measure of the extent to which the electrode reaction is affected by the polar effects of the substituents, whereas σ_*x*_ is a measure of the electronic effect that a substituent has on a molecule, and thus the redox potential in this case. The average of the sum of σ_m−*x*_ and σ_p−*x*_ [29], i.e., (σ_m−*x*_ + σ_p−*x*_)/2, was used in place of the total polar substituent constant σ_*x*_ in the Hammett plots, as recommended for the quinoxaline system [30] (Figure 4). The results observed are consistent with facilitation of reduction by a positive charge at the electroactive site [28], and are in agreement with previous studies of the electrochemical properties of quinoxaline-di-*N*-oxides [10,17,18,19,25,26,28].

A voltammetric wave representing the reduction of the second *N*-oxide functionality was observed for all derivatives studied, with the exception of **3c**, at potentials between −1.97 and −2.6 V. This process was irreversible for each of the quinoxaline derivatives. Additional irreversible reduction waves were observed at more negative potentials for compound **1a** at −2.5 (sh), −2.7 (sh), and −2.829 V. The last reduction observed appeared close to background reduction. The currents for these processes would indicate one-electron reductions in each case. However, these processes were not studied in further detail.

Compounds **1e**, **2e**, **3e** and **4e** possess a nitro group in the *para* position of the 3-aryl group. In each case, a quasireversible reduction process was observed between the two voltammetric waves representing the reductions of the *N*-oxide moieties, with half-wave potentials ranging from −1.516 to −1.563 V. ΔE_p_ and E_pc_ − E_1/2_ values at 100 mV/s for this wave ranged from 0.063 to 0.096 V and −0.031 to −0.048 V, respectively. In addition, the i_pa_/i_pc_ ratios for these derivatives at 100 mV/s were between 0.6 and 0.7. Comparison of cathodic peak currents for this process to those for the first *N*-oxide reduction, as well as using ΔE_p_ and E_pc_ − E_1/2_ for estimates, indicates that this process involves one electron. This wave may be attributed to reduction of the nitro group. Previous investigations into the electrochemical properties of the nitro group have shown that this functional group undergoes one electron reduction to form a radical anion in aprotic media [31,32,33,34]. It seems reasonable to assume that a nitro radical anion was formed during the reductions of these derivatives as well.

### 2.2. Preliminary Computational Study

The computational half-cell potentials for molecules **1a**–**1e** from Table 1 were calculated using Guassian 09 and are listed in Table 4.

**Figure 5:**
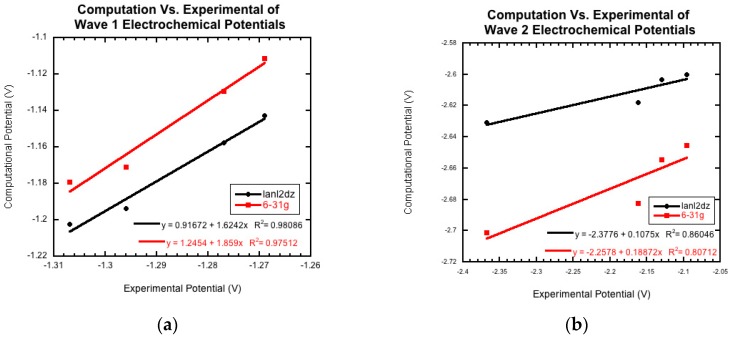
The computationally derived reduction potentials for (**a**) wave 1 (1st *N*-oxide reduction) and (**b**) wave 2 (2nd *N*-oxide reduction) compared to the experimentally measured data demonstrates a strong correlation. The basis set for lanl2dz, shown in black, can be compared to 6-31g, shown in red.

The optimization energy, thermal correction factor, and solvation energy were calculated for the first wave with the extra electron of the radical anion on both the carbon attached to the benzene ring and the carbon attached to the cyano group. The energies and thermal correction factors were found to be identical regardless of which of these carbon atoms the radical was located on (results not shown, manuscript in preparation). This result seems to indicate that the extra electron isn’t isolated to a single carbon, but instead is in some sort of resonance, as shown in Figure 3. Future computational work will investigate this observation further. For the remainder of this paper, we will only present the calculations with the radical on the carbon neighboring the cyano group. In Table 5, the computational half-cell potentials are compared to the ferrocene/ferrocinium (Fc/Fc^+^) redox couple, firstly with the standard hydrogen half-cell potential set to zero, and secondly with the ferrocene/ferrocinium reduction potential set to zero. These half-cell potentials are analyzed relative to ferrocene to provide a direct comparison to the experimental half wave potentials (E_1/2_). For derivatives **1a**–**1d**, while the computational and experimental values do not agree quantitatively, they have a strong qualitative agreement for the first and second reduction waves. Figure 5a compares the two computational basis set calculations, lanld2z (black) and 6-31g (red), to the experimental reduction potentials (E_1/2_) for wave 1. For both basis sets, there is a strong positive correlation. Figure 5b shows the comparison of the two computational basis set calculations, lanl2dz (black) and 6-31g (red), compared to the experimental peak potentials (E_pc_) for wave 2. While there is still good qualitative agreement, the correlation between the computational and experimental data decreased compared to wave 1. For the second wave, the computational values could demonstrate more accurate predictions than those generated electrochemically since this wave was found to be irreversible for all derivatives.

Molecule **1e** was not included in Figure 5. This nitro group-containing derivative displayed drastically different calculated potentials from those of derivatives **1a**–**1d** and from the experimental value. The origin of these differences will be investigated in future computational research.

### 2.3. Reduction Potentials versus Cytotoxicity

**Figure 6:**
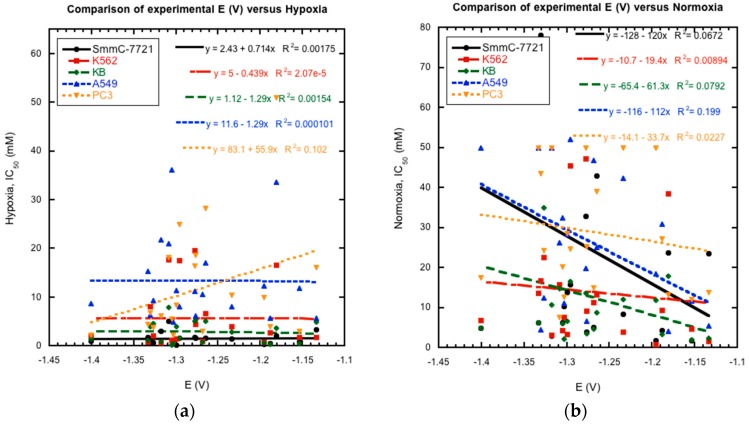
The comparison of experimentally measured reduction potentials for wave 1 (1st *N*-oxide reduction) to the previously reported IC_50_ (μM) for five cancer cell lines under (**a**) hypoxic and (**b**) normoxic conditions clearly shows no correlation. Hypoxia = 3% oxygen, Normoxia = 20% oxygen.

Previous studies of substituted quinoxaline di-*N*-oxides have demonstrated a link between reduction potential and certain biological activities, i.e., the compounds with higher activities generally have less negative reduction potentials and are easier to reduce [10,17,18,19]. Thus, possible links between reduction potential and anti-tumor activity were investigated for the current series of compounds. Comparison of their reported cytotoxicity against cancer cell lines in hypoxia and normoxia [21] versus their measured E_1/2_ values as a whole does not show a clear and direct correlation between activity and reduction potential (Figure 6). Plots of reduction potential versus cytotoxicity as a whole show no clear patterns. Comparison of smaller subsets within the data also is inconclusive. For example, derivatives **4a** and **4b** were shown to possess better hypoxic activity against cancer cell lines than the un-substituted derivatives **1a** and **1b** [21]. And the former are also more easily reduced by over 100 mV. However, derivatives **1e** and **2e** are more easily reduced than derivatives **1c** and **2c**, respectively, but have lower hypoxic activities. In addition, the most potent compound with the highest reported activities, **2c**, was not the most easily reduced derivative. These results do not rule out bioreduction in the mechanism of action of these compounds against cancer cells. However, they indicate that other factors besides bioreduction may play a more important role for the in vivo mechanism of action of these compounds, such as metabolism, stereochemistry, membrane permeability, bioactivation, DNA binding, and diffusion [35,36].

## 3. Materials and Methods

### 3.1. Chemical Synthesis

The quinoxaline derivatives studied in this paper were prepared and characterized as reported previously [21]. The structures of these test compounds are shown in Table 1.

### 3.2. Electrochemistry

All reagents used in this study were obtained in the highest purity available commercially and used as received. All solutions were prepared in dimethylformamide (DMF, Fisher Scientific, Waltham, MA, USA) with tetrabutylammonium perchlorate (TBAP, Aldrich Chemical Company, Milwaukee, WI, USA) serving as the supporting electrolyte. Test solutions contained 1.0 mM of the corresponding quinoxaline derivative and 0.1 M TBAP. Ferrocene (Fc, Sigma Aldrich, St. Louis, MO, USA) was added to each test solution following completion of the electrochemical measurements of the test compound, and used as an internal reference redox system [37] in order to account for daily variations in the reference electrode and liquid junction potentials. All potentials in this study are reported versus the ferrocene/ferrocinium (Fc/Fc^+^) redox couple:

E_pc_,_SRE_ − E_1/2,Fc/Fc_ + or E_1/2_,_SRE_ − E_1/2,Fc/Fc_^+^ (1)

Half-Wave potentials (E_1/2_) for ferrocene ranged from 0.0155 V to 0.0265 V during the course of this study. Cyclic voltammetric experiments were carried out at room temperature under an inert dinitrogen atmosphere (prepurified, Air Gas Mid-America Region, Bowling Green, KY, USA). Test solutions were deaerated for 15 min prior to obtaining the electrochemical data. A 620 Electrochemistry Analyzer (CH Instruments, Austin, TX, USA) was used for all electrochemical measurements. Solution resistance was uncompensated. A standard three electrode cell, consisting of a Pt-disk (1.6 mm diameter) working electrode, a Pt-wire auxilliary electrode, and a Ag/AgNO_3_ (0.1 M in acetonitrile) reference electrode, was used. Scan rates ranged from 0.05 V/s to 1 V/s. Half-Wave potentials were calculated using the following equation [24]: E_1/2_ = (E_pa_ + E_pc_)/2. For the first derivative cyclic voltammograms, E_p_ values were measured at the points where the derivative curves crossed the baseline [24]. Peak currents were measured from the extrapolated baselines for both the cathodic and anodic processes [23].

### 3.3. Computational Chemistry

**Figure 7:**
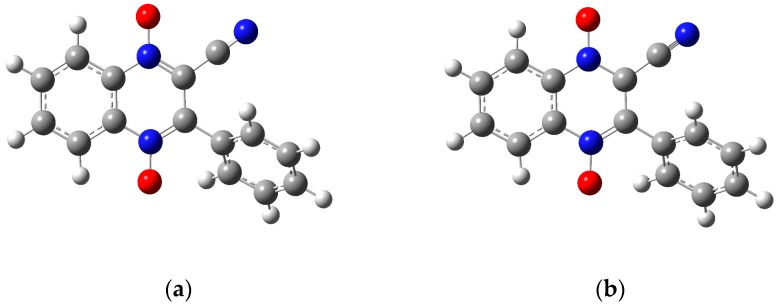

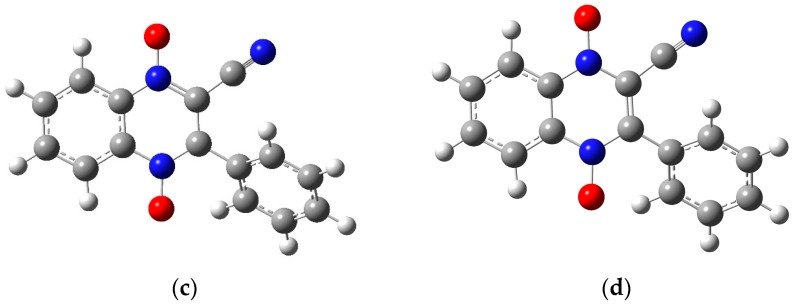
The optimized structures for molecule **1a** using the 6-31g basis set: (**a**) the neutral molecule, (**b**) the anion product from wave 1 with the radical on the carbon attached to the cyano group, (**c**) the anion product of wave 1 with the radical on the carbon attached to the benzene ring, and (**d**) the dianion product of wave 2. Figures created in GaussView 5 [39] and optimized in Gaussian 09 [38].

**Figure 8:**
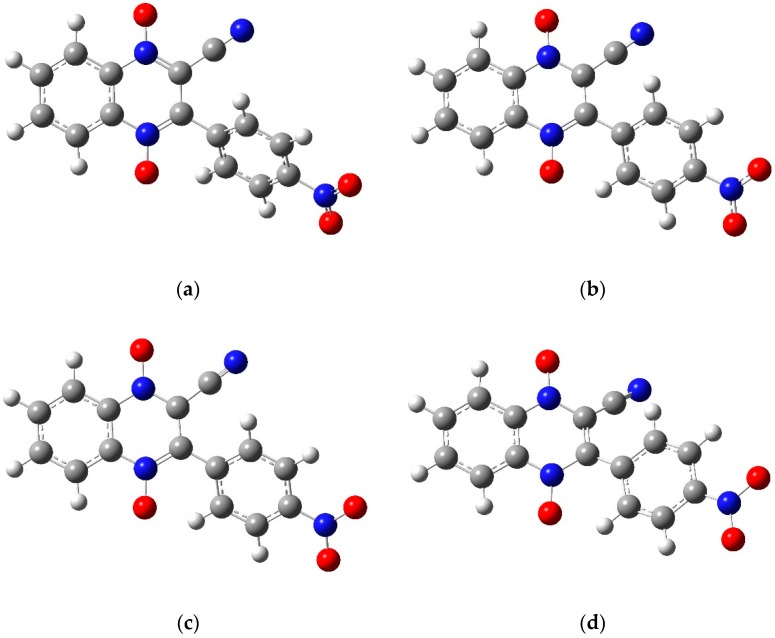
The optimized structures for molecule **1e** using the 6-31g basis set: (**a**) the neutral molecule, (**b**) the anion product of wave 1 with the radical on the carbon attached to the cyano group, (**c**) the dianion product of the NO wave with radicals on the carbon attached to the cyano and on nitro group, and (**d**) the trianion product of wave 2. Figures created in GaussView 5 [39] and optimized in Gaussian 09 [38].

Gaussian 9.0 was used to model the 3-aryl-quinoxaline-2-carbonitrile 1,4-di-*N*-oxide derivatives **1a**–**1e** (Table 1) [38]. For **1a**–**1d**, the neutral molecule, the radical anion and dianion structures were drawn in GaussView 5 [39]. Figure 7 shows the neutral molecule, two possible radical anions and the dianion structures used for molecule **1a**. For molecule **1e**, the neutral molecule, the anion with the radical on the carbon attached to the cyano group, the dianion with radicals on the carbon attached to the cyano group and on the nitro group and trianion structures are shown in Figure 8. Each structure was optimized using two common basis sets, lanl2dz and 6-31g, to determine the lowest energy conformation. Next, a frequency calculation was performed to correct for thermal artifacts present in the program. The energy of each structure was found by summing the optimization energy and the thermal correction factor. The change in Gibbs Free Energy from the addition of an electron was calculated by subtracting the free energy of the appropriate structures. Lastly, an energy calculation was used to obtain an energy of each molecule solvated in *N*,*N*-dimethylformamide. The change in Gibbs Free Energy of solvation (ΔG_solv_) was found by subtracting the energy of the molecule from the solvated energy of the molecule [22,40].

**Figure 9:**
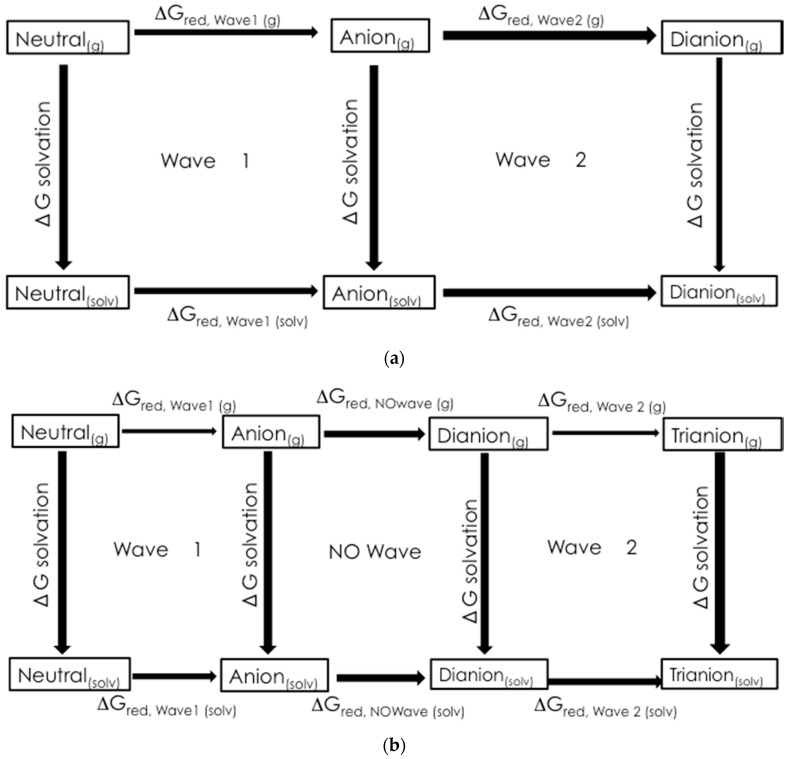
The thermodynamic cycles used to calculate ΔG_red_: (**a**) wave 1 (1st *N*-oxide reduction) and wave 2 (2nd *N*-oxide reduction) for **1a**–**1d** and (**b**) wave 1 (1st *N*-oxide reduction), NO wave (nitro group reduction) and wave 2 (2nd *N*-oxide reduction) for **1e**.

Figure 9 shows the thermodynamical cycles used to calculate the change in Gibbs Free Energy associated with the reduction of wave 1 and wave 2, ΔG_red_,_wave 1(solv)_ and ΔG_red_,_wave 2(solv)_, respectively. For wave 1:

ΔG_red,wave 1(solv)_ = −ΔG_solv,n_ + ΔG_red,wave 1_(g) + −ΔG_solv,r_ (2)

where ΔG_solv,n_ is the change in Gibbs Free Energy of solvation of the neutral molecule, ΔG_red,wave 1_(g) is the change in Gibbs Free Energy of the reduction in the gas phase, and ΔG_solv,r_ is the change in Gibbs Free Energy of solvation for the radical anion.

The ΔG_red_ values were used to calculate the half-cell reduction potentials for comparison to the experimental data. The half-cell potentials were referenced to the ferrocene/ferrocinium half-cell.

## Figures and Tables

**Table 1:**
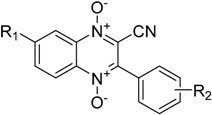
Structures of the 3-aryl-quinoxaline-2-carbonitrile 1,4-di-*N*-oxide derivatives [21].

Compound	R_1_	R_2_
**1a**	H	H
**1b**	H	3-CH_3_
**1c**	H	3-Cl
**1d**	H	4-Br
**1e**	H	4-NO_2_
**2a**	CH_3_	H
**2b**	CH_3_	3-CH_3_
**2c**	CH_3_	3-Cl
**2d**	CH_3_	4-Br
**2e**	CH_3_	4-NO_2_
**3a**	OCH_3_	H
**3b**	OCH_3_	3-CH_3_
**3c**	OCH_3_	3-Cl
**3d**	OCH_3_	4-Br
**3e**	OCH_3_	4-NO_2_
**4a**	Cl	H
**4b**	Cl	3-CH_3_
**4c**	Cl	3-Cl
**4d**	Cl	4-Br
**4e**	Cl	4-NO_2_

**Table 2:** Cyclic voltammetric data of the 3-aryl-quinoxaline-2-carbonitrile 1,4-di-*N*-oxide derivatives ^a^.

	1st *N*-oxide Reduction					Nitro Group Reduction					2nd *N*-oxide Reduction ^b^	
Compound	E_1/2_ (V)	ΔE_p_ (V)	E_pc_ − E_1/2_ (V)	i_pc_ (μA)	i_pa_/i_pc_	E_1/2_ (V)	ΔE_p_ (V)	E_pc_ − E_1/2_ (V)	i_pc_ (μA)	i_pa_/i_pc_	E_pc_ (V)	i_pc_ (μA)
**1a**	−1.296	0.076	−0.038	4.883	0.918						−2.163	3.869
**1b**	−1.309	0.082	−0.041	2.135	0.348						−2.56 (sh) ^c^	
**1c**	−1.269	0.070	−0.035	2.846	0.761						−2.097	3.753
**1d**	−1.278	0.069	−0.035	1.177	0.689						−2.06 (sh) ^c^	
**1e**	−1.234	0.103	−0.051	3.220	0.297	−1.518	0.088	−0.044	3.826	0.630	−2.306	3.808
**2a**	−1.327	0.097	−0.049	3.488	0.830						−2.310	1.501
**2b**	−1.318	0.103	−0.051	3.375	0.847						−2.377	3.395
**2c**	−1.303	0.089	−0.045	4.119	0.835						−2.125	2.020
**2d**	−1.305	0.099	−0.050	4.411	0.864						−2.326	5.656
**2e**	−1.265	0.083	−0.042	5.261	0.270	−1.539	0.076	−0.038	5.280	0.641	−2.352	7.525
**3a**	−1.331	0.077	−0.039	3.675	0.798						−2.166	2.518
**3b**	−1.333	0.079	−0.039	4.253	0.768						−2.216	3.049
**3c**	−1.401 ^b^			7.460								
**3d**	−1.300	0.075	−0.037	4.601	0.767						−1.995	3.303
**3e**	−1.277	0.095	−0.047	3.392	0.239	−1.566	0.096	−0.044	2.546	0.699	−2.372	3.816
**4a**	−1.188	0.076	−0.038	4.237	0.871						−1.973	3.650
**4b**	−1.196	0.093	−0.047	3.753	0.859						−2.115	2.118
**4c**	−1.154	0.129	−0.065	2.899	0.841						−2.080	1.342
**4d**	−1.181	0.107	−0.053	4.103	0.930						−2.132	2.866
**4e**	−1.134	0.072	−0.036	3.071	0.483	−1.514	0.063	−0.031	3.775	0.641	−2.141	3.904

^a^ Substrate, 1.0 mM; TBAP, 0.10 M; DMF; Pt working electrode; Ag/AgNO_3_ reference electrode; Pt wire counter electrode; 100 mV/s; room temperature; E vs. (Fc/Fc^+^)/V; currents reported in μA; voltammograms recorded with a CH Instruments Model 620 Electrochemistry Analyzer. ^b^ Irreversible. ^c^ Shoulder.

**Table 3:** First derivative voltammetric data of the 3-aryl-quinoxaline-2-carbonitrile 1,4-di-*N*-oxide derivatives ^a,b^.

	1st *N*-oxide Reduction			Nitro Group Reduction				2nd *N*-oxide Reduction ^c^
Compound	E_pc_ (V)	E_pa_ (V)	E_1/2_ (V)	E_pc_ (V)	E_pa_ (V)	E_1/2_ (V)	ΔE_p_ (V)	E_pc_ (V)
**1a**	−1.335	−1.256	−1.296					−2.162
**1b**	−1.350	−1.264	−1.307					−2.368
**1c**	−1.305	−1.232	−1.269					−2.096
**1d**	−1.314	−1.240	−1.277					−2.130
**1e**	−1.287	−1.179	−1.233	−1.574	−1.467	−1.521	0.107	−2.309
**2a**	−1.375	−1.276	−1.326					−2.308
**2b**	−1.369	−1.266	−1.318					−2.366
**2c**	−1.348	−1.258	−1.303					−2.122
**2d**	−1.356	−1.253	−1.305					−2.279
**2e**	−1.308	−1.221	−1.265	−1.577	−1.499	−1.538	0.078	−2.355
**3a**	−1.372	−1.290	−1.331					−2.168
**3b**	−1.374	−1.292	−1.332					−2.216
**3c**	−1.402		−1.402 ^c^					−2.466
**3d**	−1.339	−1.26	−1.299					−1.996
**3e**	−1.328	−1.226	−1.277	−1.612	−1.510	−1.562	0.101	−2.371
**4a**	−1.227	−1.148	−1.188					−1.974
**4b**	−1.243	−1.146	−1.195					−2.114
**4c**	−1.219	−1.086	−1.154					−2.082
**4d**	−1.236	−1.126	−1.181					−2.130
**4e**	−1.172	−1.094	−1.133	−1.544	−1.476	−1.510	0.068	−2.142

^a^ Substrate, 1.0 mM; TBAP, 0.10 M; DMF; Pt working electrode; Ag/AgNO_3_ reference electrode; Pt wire counter electrode; 100 mV/s; room temperature; E vs. (Fc/Fc^+^)/V; currents reported in μA; voltammograms recorded with a CH Instruments Model 620 Electrochemistry Analyzer. ^b^ E_pc_ and E_pa_ determined at the point where the derivative curve crosses the baseline [24]. ^c^ Irreversible.

**Table 4:** The half-cell reaction potentials in volts for the 1st *N*-oxide, nitro group and 2nd *N*-oxide reductions calculated using the lanl2dz and 6-31g basis sets in Gausian 09.

Compound	1st *N*-oxide Reduction		Nitro Group Reduction		2nd *N*-oxide Reduction	
	Lanl2dz	6-31g	Lanl2dz	6-31g	Lanl2dz	6-31g
**1a**	3.6006	3.2891			2.1763	1.7774
**1b**	3.5920	3.2807			2.1636	1.7586
**1c**	3.6516	3.3488			2.1944	1.8146
**1d**	3.6364	3.3307			2.1910	1.8055
**1e**	3.8605	3.5518	2.6292	2.2985	2.9601	2.6072

**Table 5:** The reduction potentials in volts for the 1st *N*-oxide, nitro group and 2nd *N*-oxide reductions versus ferrocene, calculated using the lanl2dz and 6-31g basis sets in Gausian 09.

	1st *N*-oxide Reduction		Nitro Group Reduction		2nd *N*-oxide Reduction	
**Hydrogen Half−Cell Reduction Set to Zero**						
**Compound**	**Lanl2dz**	**6-31g**	**Lanl2dz**	**6-31g**	**Lanl2dz**	**6-31g**
**1a**	−1.9136	−1.8908			−3.3379	−3.4025
**1b**	−1.9223	−1.8993			−3.3507	−3.4213
**1c**	−1.8626	−1.8312			−3.3199	−3.3654
**1d**	−1.8778	−1.8492			−3.3233	−3.3744
**1e**	−1.6538	−1.6281	−2.8851	−2.8814	−2.5542	−2.5728
**Ferrocene Half−Cell Reduction Set to Zero**						
**Compound**	**Lanl2dz**	**6-31g**	**Lanl2dz**	**6-31g**	**Lanl2dz**	**6-31g**
**1a**	−1.1936	−1.1708			−2.6179	−2.6825
**1b**	−1.2023	−1.1793			−2.6307	−2.7013
**1c**	−1.1426	−1.1112			−2.5999	−2.6454
**1d**	−1.1578	−1.1292			−2.6033	−2.6544
**1e**	−0.9338	−0.9081	−2.1651	−2.1614	−1.8342	−1.8528

## Abbreviations

Dimethylformamide	DMF
half-wave potential	E_1/2_
anodic peak potential	E_pa_
cathodic peak potential	E_pc_
difference in peak potentials	ΔE_p_
anodic peak current	i_pa_
cathodic peak current	i_pc_
ratio of peak currents	i_pa_/i_pc_
reaction constant	ρ_π,R_
total polar substituent constant	σ_*x*_
substituent constant for meta substituent	σ_m−*x*_
substituent constant for para substituent	σ_p−*x*_
